# West Nile Virus (WNV) Infection-Associated Acute Flaccid Paralysis With Ophthalmoplegia

**DOI:** 10.7759/cureus.38137

**Published:** 2023-04-26

**Authors:** Srikanth Adidam Venkata, Narek Hakobyan, David P Lerner, Ramaswami Sundar, Arthur Kay

**Affiliations:** 1 Internal Medicine, Brookdale University Hospital Medical Center, New York City, USA; 2 Neurology, Brookdale University Hospital Medical Center, New York City, USA

**Keywords:** alcohol use, encephalopathy, flaccid paralysis, bilateral ophthalmoplegia, neuroinvasive west nile virus

## Abstract

Infection with West Nile virus (WNV) is often characterized by a mild febrile illness, but it can progress to meningitis, encephalitis, flaccid paralysis, and respiratory failure. The neuro-ophthalmological manifestations of this disease are uncommonly discussed. This case describes a 49-year-old undomiciled male who developed WNV flaccid paralysis with ophthalmoplegia. His symptoms began with difficulty in walking and progressed over several days to flaccid paralysis and ophthalmoplegia. Cerebrospinal fluid was positive for WNV immunoglobulin M antibodies and electromyography demonstrated acute denervation in several muscle groups. This is an unusual case of neuro-invasive WNV presenting with flaccid paralysis and ophthalmoplegia.

## Introduction

The West Nile virus (WNV) is a member of the Japanese encephalitis serogroup of genus *Flavivirus*, family Flaviviridae, which has an incubation period of 2-14 days. The incidental hosts include birds and mosquitoes, and humans are the “dead-end” hosts [1]. WNV can present as a variety of symptoms, but most commonly as West Nile fever, which is an acute, limited flu-like illness characterized by fatigue, myalgia, arthralgia and a red maculopapular rash. In a small percentage of cases, WNV can cause neuro-invasive diseases such as meningitis, encephalitis, acute flaccid paralysis, or cranial neuropathies such as facial nerve paresis, but rarely ocular symptoms. In most cases, WNV is diagnosed through cerebrospinal fluid (CSF) analysis for WNV IgM antibodies, which has a specificity of 99.3%, and electromyography, which indicates acute muscle denervation due to anterior horn cell involvement. Neuro-invasive WNV disease may also be diagnosed through magnetic resonance imaging (MRI) of the spinal cord showing abnormally increased T2 signaling in the anterior gray matter [2].

## Case presentation

A 46-year-old male, former smoker and with alcohol use disorder, presented to the emergency department (ED) with gradually progressive paraparesis over six days. Initially, he was ambulatory, but as the weakness progressed, he suffered a fall prompting his presentation to the ED. On evaluation, the patient also commented on mild dysphagia and weakness in his left arm. Prior to the onset of symptoms, he recalled having a flu-like illness but denied recent travel, diarrhea, vaccinations, fever, chills, or weight loss. Vital signs in the ED were remarkable for blood pressure at 129/81 mmHg, heart rate 98 beats per minute, afebrile state, and respiratory rate of 20 breaths per minute. Relevant laboratory test details are included in Table 1.

**Table 1:** Relevant serum laboratory results

Laboratory test	Results	Reference range
Potassium	2.8 mmol/L	3.5-5.1 mmol/L
Sodium	118 mmol/L	133-145 mmol/L
Calcium	7.8 mg/dL	8.4-10.5 mg/dL

The neurology team evaluated the patient in the ED. His examination demonstrated generalized weakness in all extremities due to combined hypokalemia, hypocalcemia, and hyponatremia secondary to alcohol misuse. Despite electrolyte correction, there was no improvement in the weakness. Over the course of three days, he developed worsening dysphagia, quadriparesis and neck flexion weakness. Initially, the patient did not experience any difficulty in breathing, but his weakness and respiratory difficulties gradually worsened necessitating intubation and invasive ventilatory support.

Repeat neurology examination in the intensive care unit demonstrated ophthalmoplegia, asymmetric reflexes, and quadriplegia raising suspicion of a Miller-Fisher variant of Guillain-Barré syndrome versus Bickerstaff brainstem encephalitis. In addition to administering intravenous immunoglobulins, cerebrospinal fluid was collected for analysis. CSF analysis results are shown in Table 2.

**Table 2:** Cerebrospinal fluid analysis results IgM, immunoglobulin M

Laboratory test	Results	Reference range
White blood cell count	31 cells per microliter	0-5 cells per microliter
Red blood cell count	6 cells per microliter	Negative
Protein	143 mg/dL	25-45 mg/dL
Glucose	67 mg/dL	40-70 mg/dL
West Nile virus IgM	Positive	Negative

**Figure 1:**
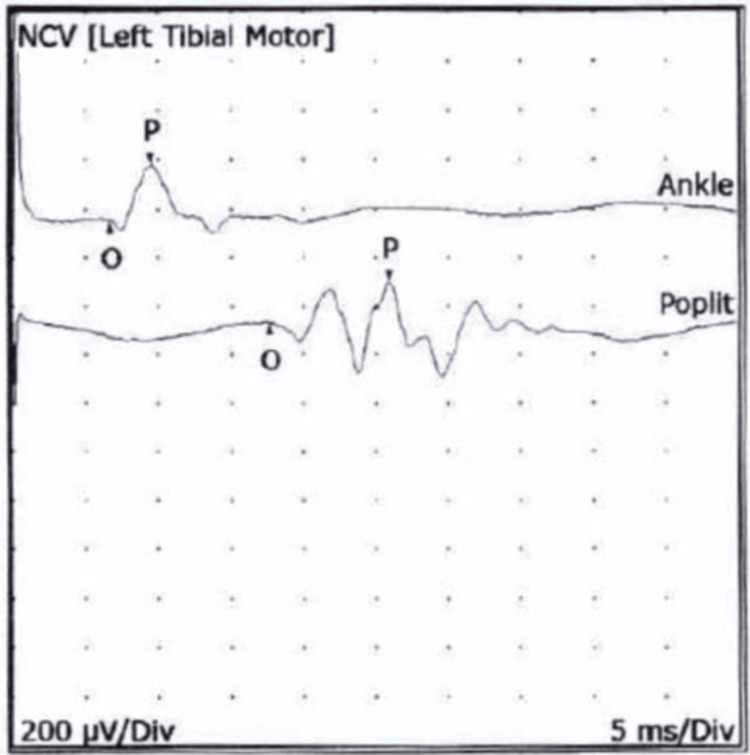
An electroneurogram showing the temporal dispersion of motor evoked potentials NCV, nerve conduction velocity

Unfortunately, immune globulin therapy did not prove effective, leading to the initiation of plasma exchange therapy. An electromyogram (EMG) was performed that showed findings that were consistent with an axonal neuropathy, predominantly motor, and affecting the upper and lower extremities. Testing of the left tibial motor nerve showed a distal latency of 6.7 milliseconds, compound muscle activation potential amplitude of 200 microvolts, and motor conduction velocity (MCV) 39 meters per second. In addition to the left tibial motor nerve, bilateral median and ulnar motor nerves, bilateral peroneal motor nerves and right tibial motor nerve were tested. Bilateral median sensory nerves, bilateral ulnar sensory nerves, bilateral superficial peroneal sensory nerves and bilateral sural sensory nerves were also tested. On review of the electroneurogram, minor amplitude abnormalities were seen (Figure 1).

**Figure 2:**
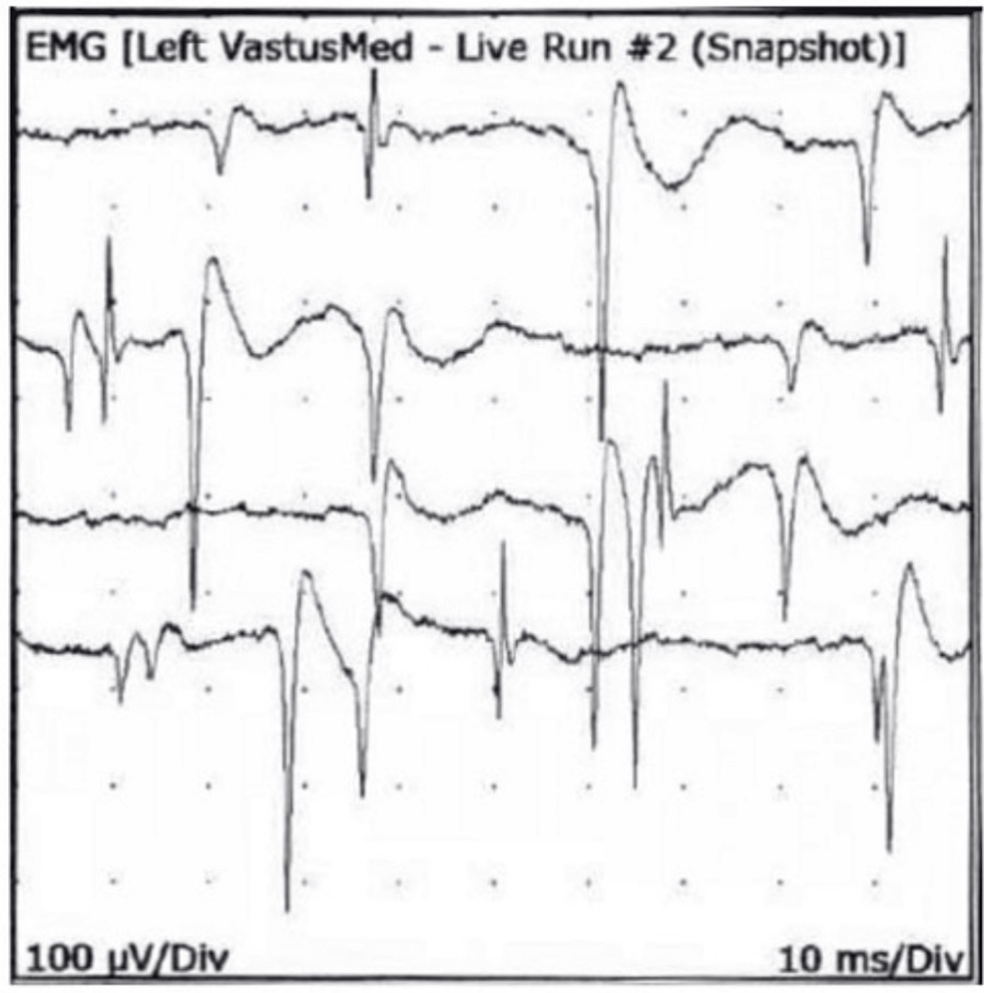
An electromyogram, done when the muscle is resting, showing fibrillary potentials and positive sharp waves

Fibrillary potentials were also noted indicating demyelination and axonal involvement (Figure 2).

**Figure 3:**
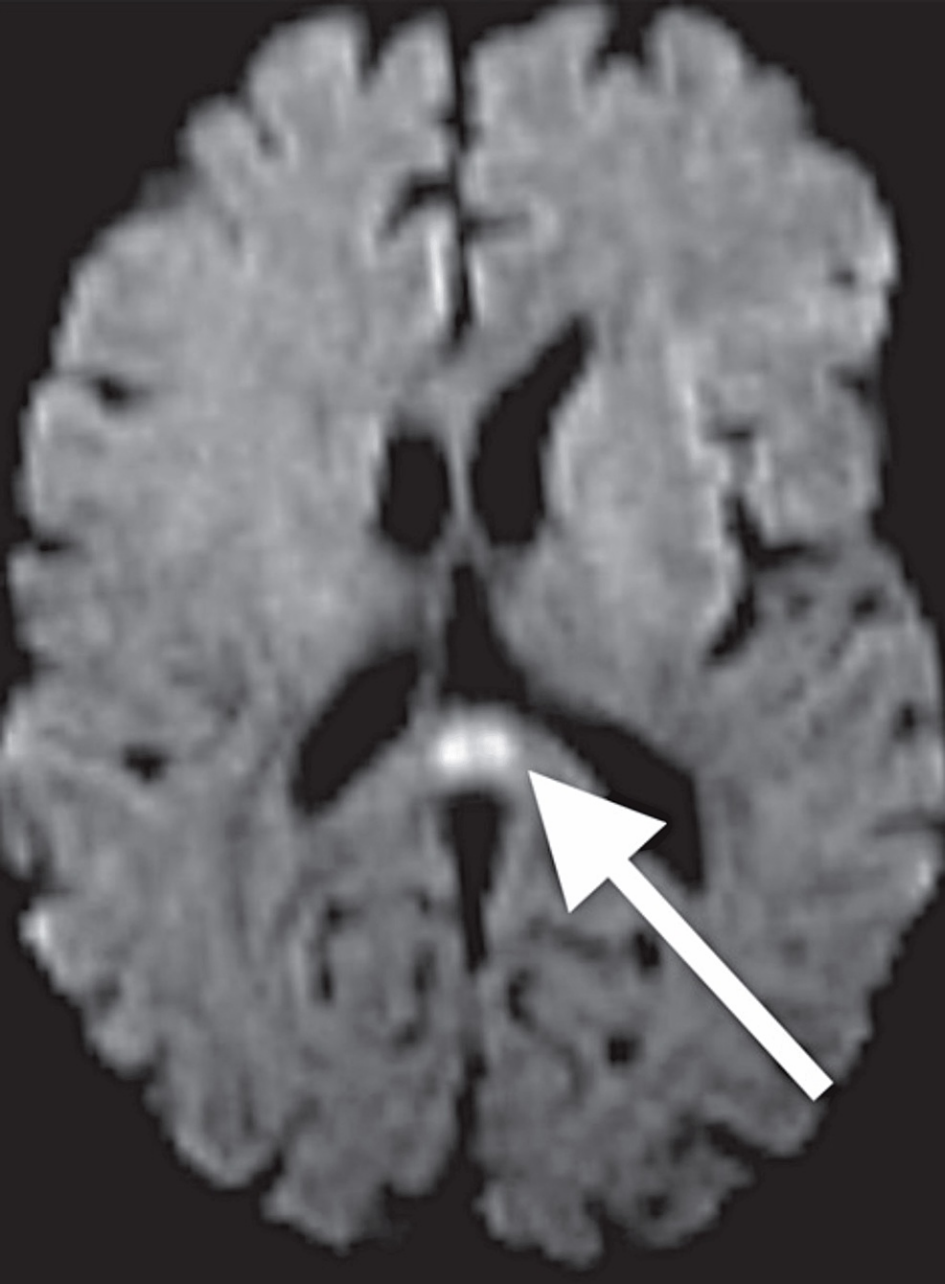
MRI of the brain showing diffusion weighted imaging restricted diffusion of the splenium of the corpus callosum

Magnetic resonance imaging (MRI) of the brain showed diffusion restriction on diffusion weighted imaging (DWI) of the splenium of the corpus callosum without apparent diffusion coefficient enhancement indicating cytotoxic injury (Figure 3).

**Figure 4:**
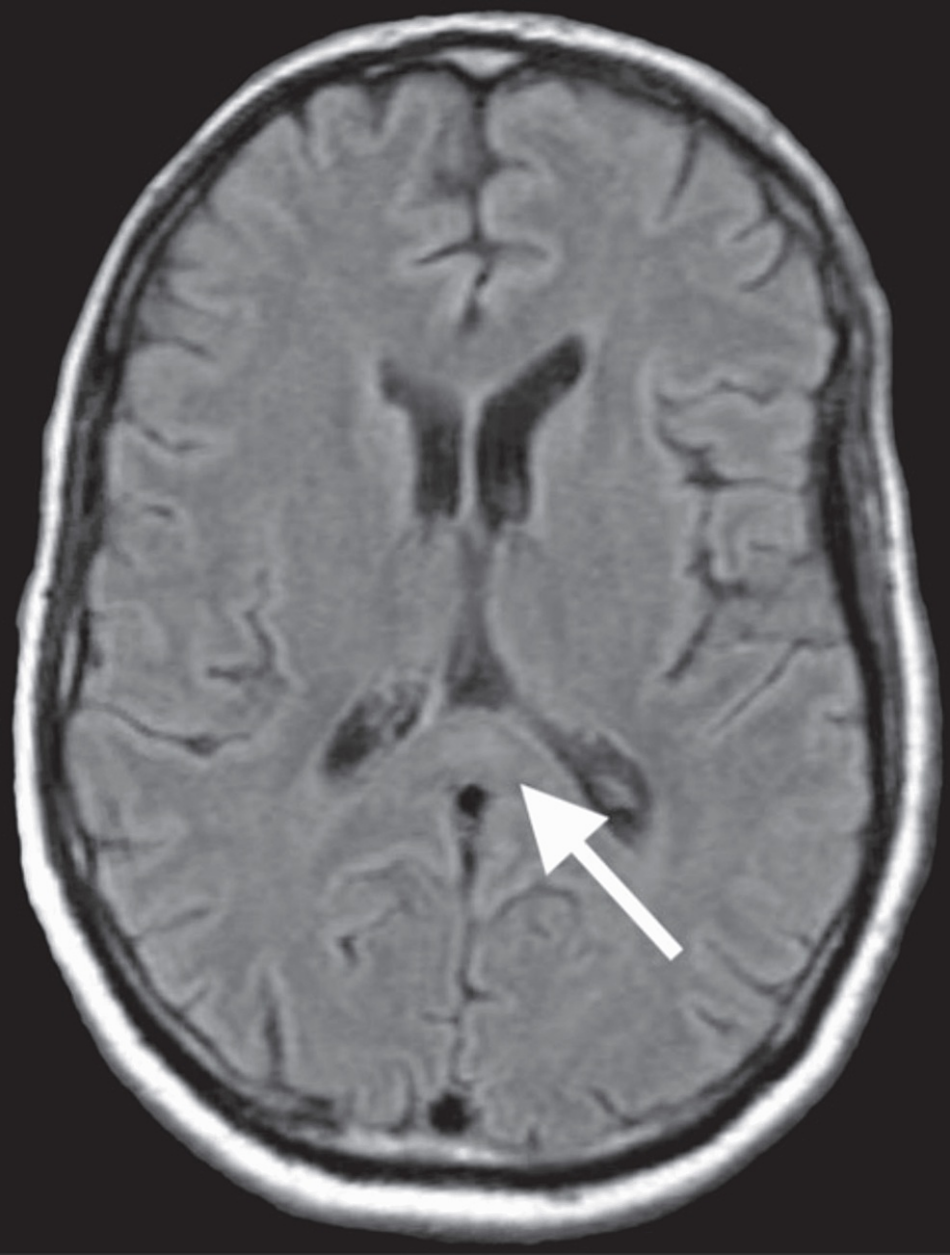
MRI of the brain showing T2/fluid attenuated inversion recovery signal hyperintensity of the splenium of the corpus callosum

Associated T2/fluid attenuated inversion recovery (FLAIR) signal hyperintensity demonstrated hyperintensity of the corpus callosum (Figure 4). MRI of the cervical, thoracic, and lumbar spine was normal.

With intubation, supportive treatment and intravenous immunoglobulins and plasma exchange therapy, he experienced resolution of his ophthalmoplegia but only minimal improvement in his quadriplegia with flexion of his right fingers and a slight extension of the right elbow, as well as trace movements of his left toes. Ultimately, he underwent a tracheostomy and a percutaneous endoscopic gastrostomy (PEG) tube placement as he required prolonged ventilation, artificial feeding and was transferred to rehabilitation services.

## Discussion

WNV is a member of the Flaviviridae family and is part of the Japanese encephalitis serocomplex. Despite being endemic in Africa and the Middle East, there was an outbreak of the disease in New York, USA, in 1999. In spite of its initial presentation as a nonspecific febrile illness that is typically classified as West Nile fever, it is becoming recognized as a common cause of viral encephalitis in the United States. Several factors contribute to the development of neuro-invasive diseases, including advancing age, male gender, and comorbid conditions, such as diabetes and immunosuppression. Flaccid paralysis and degree of motor neuron dysfunction on the EMG are associated with worse prognosis [3]. Recovery may take months or years, and most patients may never achieve their baseline [4]. The MRI of the brain in this case was not considered to be related to his WNV infection.

In addition to flaccid paralysis, WNV encephalitis with ophthalmic features is characterized by optic neuritis and chorioretinitis [5]. The symptoms of our patient with West Nile neuro-invasive disease, flaccid paralysis with ophthalmoplegia, are rare in the literature.

The most common cranial nerve affected with WNV-associated ophthalmoplegia is the abducens nerve, sometimes in conjunction with facial nerve paresis. Among patients with West Nile encephalitis, pathology reports indicate that the most severe inflammation occurs in the medulla and cranial nerve roots and could explain such presentations [6]. While not the hallmark symptoms of West Nile virus infection, encephalitis with ophthalmoplegia and ataxia are the main components in the description of the anti-ganglioside Q1b (anti-GQ1b) antibody group of diseases. As a result of this clinical overlap, neuro-invasive WNV has been initially diagnosed as Bickerstaff brainstem encephalitis or a Guillain-Barré syndrome variant [7].

Although rarely documented, WNV-associated flaccid paralysis with ophthalmoplegia follows the same pattern of disease as other mosquito-transmitted Flaviviridae infections. There is a relatively low incidence of eastern and western equine encephalitis, St. Louis encephalitis, and Japanese encephalitis in the United States, but they are occasionally associated with optic neuritis, abducens, and facial palsies [8]. When it comes to differentiating WNV-associated neuro-invasive disease from other encephalitic viruses, flaccid paralysis should be considered a defining feature [9]. The risk of developing neuro-invasive WNV disease was found to be increased among male patients and those older than 64 years of age. A history of alcohol abuse has been independently associated with more severe WNV disease and adverse outcomes, including death and severe encephalitis [10].

## Conclusions

A tropical illness, the WNV has historically been associated with New York, and is on the rise in North America. Physician vigilance is warranted in cases of ascending paralysis, particularly among undomiciled, older males with alcohol abuse history and immunosuppression. CSF analysis and EMG can provide diagnostic evidence for WNV neuro-invasive disease. It is rare for an individual to suffer from ophthalmoplegia with flaccid paralysis as a result of West Nile virus; however, physicians should be aware of these symptoms in such cases in order to help differentiate between West Nile virus infection and other neuroinflammatory diseases.
